# (*E*)-2-Chloro-*N*′-(2-hydr­oxy-1-naphthyl­methyl­ene)benzohydrazide

**DOI:** 10.1107/S1600536808031371

**Published:** 2008-10-04

**Authors:** Feng Qiu, Li-Mei Zhao

**Affiliations:** a Department of Pharmacy, The Sheng Jing Hospital of Chinese Medical University, Shenyang 110004, People’s Republic of China

## Abstract

In the structue of the title compound, C_18_H_13_ClN_2_O_2_, a new Schiff base, the dihedral angle between the benzene and naphthyl ring system mean planes is 22.5 (2)°. The mol­ecule has an *E* configuration about the C=N bond, and an intra­molecular hydrogen bond involving the hydoxyl substituent on the naphthyl ring and the N′ atom of the hydrazide. The crystal structure is stabilized by inter­molecular N—H⋯O hydrogen bonds, forming one-dimensional chains running parallel to the *a* axis.

## Related literature

For background on Schiff base compounds, hydrazone compounds and their biological properties, see: Kucukguzel *et al.* (2006 ▶); Khattab (2005 ▶); Karthikeyan *et al.* (2006 ▶); Okabe *et al.* (1993 ▶). For bond distances, see: Allen *et al.* (1987 ▶). For related structures, see: Shan *et al.* (2008 ▶); Fun *et al.* (2008 ▶); Yang (2008 ▶); Ma *et al.* (2008 ▶); Diao, Huang *et al.* (2008 ▶); Diao, Zhen *et al.* (2008 ▶); Ejsmont *et al.* (2008 ▶).
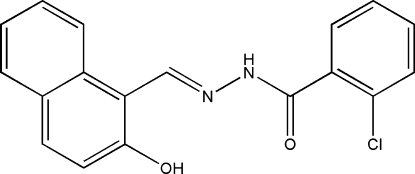


## Experimental

### 

#### Crystal data

C_18_H_13_ClN_2_O_2_
*M* *_r_* = 324.75
Monoclinic, 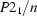

*a* = 7.2797 (14) Å
*b* = 29.148 (6) Å
*c* = 7.6889 (16) Å
β = 112.130 (3)°
*V* = 1511.3 (5) Å^3^
*Z* = 4
Mo *K*α radiation
μ = 0.26 mm^−1^
*T* = 298 (2) K
0.32 × 0.27 × 0.26 mm

#### Data collection

Bruker SMART CCD area-detector diffractometer
Absorption correction: multi-scan (*SADABS*; Bruker, 2001 ▶) *T*_min_ = 0.920, *T*_max_ = 0.934
8693 measured reflections
3255 independent reflections
2320 reflections with *I* > 2σ(*I*)
*R*_int_ = 0.036

#### Refinement

*R*[*F* ^2^ > 2σ(*F* ^2^)] = 0.044
*wR*(*F* ^2^) = 0.105
*S* = 1.03
3255 reflections
213 parameters
1 restraint
H atoms treated by a mixture of independent and constrained refinement
Δρ_max_ = 0.21 e Å^−3^
Δρ_min_ = −0.20 e Å^−3^

### 

Data collection: *SMART* (Bruker, 2007 ▶); cell refinement: *SAINT* (Bruker, 2007 ▶); data reduction: *SAINT*; program(s) used to solve structure: *SHELXTL* (Sheldrick, 2008 ▶); program(s) used to refine structure: *SHELXTL*; molecular graphics: *SHELXTL*; software used to prepare material for publication: *SHELXTL*.

## Supplementary Material

Crystal structure: contains datablocks global, I. DOI: 10.1107/S1600536808031371/su2067sup1.cif

Structure factors: contains datablocks I. DOI: 10.1107/S1600536808031371/su2067Isup2.hkl

Additional supplementary materials:  crystallographic information; 3D view; checkCIF report

## Figures and Tables

**Table 1:** Hydrogen-bond geometry (Å, °)

*D*—H⋯*A*	*D*—H	H⋯*A*	*D*⋯*A*	*D*—H⋯*A*
N2—H2⋯O2^i^	0.896 (9)	1.972 (11)	2.842 (2)	163.5 (18)
O1—H1⋯N1	0.82	1.86	2.581 (2)	146

Symmetry code: (i) 
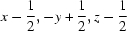
.

**Fig. 1.**
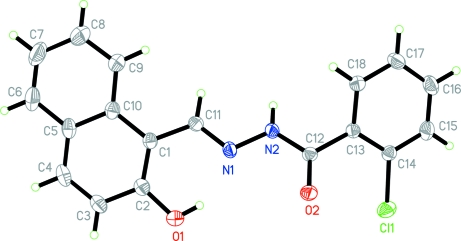
The molecular structure of compound (I) with 30% probability displacement ellipsoids for non-H atoms.

**Fig. 2.**
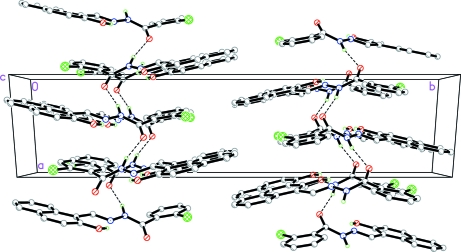
Crystal packing of compound (I) viewed along the c axis (Hydrogen bonds are shown as dashed lines).

## supplementary crystallographic information

### Comment

Hydrazones and Schiff bases have attracted much attention for their excellent biological properties, especially for their potential pharmacological and antitumor properties (Kucukguzel *et al.*, 2006; Khattab, 2005; Karthikeyan *et al.*, 2006; Okabe *et al.*, 1993). Recently, a large number of hydrazone derivatives have been prepared and structurally characterized (Shan *et al.*, 2008; Fun *et al.*, 2008; Yang, 2008; Ma *et al.*, 2008; Diao, Huang *et al.*, 2008; Diao, Zhen *et al*., 2008; Ejsmont *et al.*, 2008). As part of an ongoing study, we report herein the crystal structure of the title compound, (I).

The molecular structure of compound (I) is shown in Fig. 1. The bond dstances and angles are normal (Allen *et al.*, 1987). The dihedral angle between the phenyl and naphthyl ring mean planes is 22.5 (2)°. The compound displays an *E* configuration about the C═N bond, and an intramolecular hydrogen bond involving the hydoxyl substituent on the naphthyl ring and the N-atom of the hydrazide (Table 1). The crystal structure is stabilized by intermolecular N—H···O hydrogen bonds (Table 1), forming one-dimensional chains running parallel to the *a* axis, Fig. 2.

### Experimental

Compound (I) was prepared by dissolving 2-Hydroxy-1-naphthaldehyde (1.0 mmol, 172.3 mg) in methanol (50 ml), then 2-chlorobenzohydrazide (1.0 mmol, 170.2 mg) was added slowly and the mixture kept at reflux with continuous stirring for 3 h. When the solution was cooled to room temperature a colourless crystalline powder appeared. This was filtered off and washed with methanol three times. Recrystallization from absolute methanol yielded block-shaped single crystals suitable for X-ray analysis.

### Refinement

H-atom H2 was located in a difference Fourier map and refined isotropically, with the N–H distance restrained to 0.90 (1) Å. The other H-atoms were placed in calculated positions and treated as riding atoms: O–H = 0.82 Å, C–H = 0.93 Å, with U_iso_(H) = 1.2U_eq_(C) and 1.5U_eq_(O).

### Crystal data

**Table:**

C_18_H_13_ClN_2_O_2_	*F*(000) = 672
*M**_r_* = 324.75	*D*_x_ = 1.427 Mg m^−^^3^
Monoclinic, *P*2_1_/*n*	Mo *K*α radiation, λ = 0.71073 Å
*a* = 7.2797 (14) Å	Cell parameters from 2129 reflections
*b* = 29.148 (6) Å	θ = 2.5–25.3°
*c* = 7.6889 (16) Å	µ = 0.26 mm^−^^1^
β = 112.130 (3)°	*T* = 298 K
*V* = 1511.3 (5) Å^3^	Block, colourless
*Z* = 4	0.32 × 0.27 × 0.26 mm

### Data collection

**Table:**

Bruker SMART CCD area-detector diffractometer	3255 independent reflections
Radiation source: fine-focus sealed tube	2320 reflections with *I* > 2σ(*I*)
graphite	*R*_int_ = 0.036
ω scans	θ_max_ = 27.0°, θ_min_ = 2.8°
Absorption correction: multi-scan (SADABS; Bruker, 2001)	*h* = −9→9
*T*_min_ = 0.920, *T*_max_ = 0.935	*k* = −31→37
8693 measured reflections	*l* = −9→6

### Refinement

**Table:**

Refinement on *F*^2^	Primary atom site location: structure-invariant direct methods
Least-squares matrix: full	Secondary atom site location: difference Fourier map
*R*[*F*^2^ > 2σ(*F*^2^)] = 0.044	Hydrogen site location: inferred from neighbouring sites
*wR*(*F*^2^) = 0.105	H atoms treated by a mixture of independent and constrained refinement
*S* = 1.03	*w* = 1/[σ^2^(*F*_o_^2^) + (0.0421*P*)^2^ + 0.2242*P*] where *P* = (*F*_o_^2^ + 2*F*_c_^2^)/3
3255 reflections	(Δ/σ)_max_ < 0.001
213 parameters	Δρ_max_ = 0.21 e Å^−^^3^
1 restraint	Δρ_min_ = −0.20 e Å^−^^3^

### Special details

**Table:**

Geometry. All esds (except the esd in the dihedral angle between two l.s. planes) are estimated using the full covariance matrix. The cell esds are taken into account individually in the estimation of esds in distances, angles and torsion angles; correlations between esds in cell parameters are only used when they are defined by crystal symmetry. An approximate (isotropic) treatment of cell esds is used for estimating esds involving l.s. planes.
Refinement. Refinement of F^2^ against ALL reflections. The weighted R-factor wR and goodness of fit S are based on F^2^, conventional R-factors R are based on F, with F set to zero for negative F^2^. The threshold expression of F^2^ > 2sigma(F^2^) is used only for calculating R-factors(gt) etc. and is not relevant to the choice of reflections for refinement. R-factors based on F^2^ are statistically about twice as large as those based on F, and R- factors based on ALL data will be even larger.

### Fractional atomic coordinates and isotropic or equivalent isotropic displacement parameters (Å^2^)

**Table:**

	*x*	*y*	*z*	*U*_iso_*/*U*_eq_	
Cl1	0.90066 (8)	0.109541 (17)	0.58158 (8)	0.05120 (18)	
N1	0.9283 (2)	0.28478 (5)	0.6687 (2)	0.0368 (4)	
N2	0.8931 (2)	0.25535 (5)	0.5185 (2)	0.0368 (4)	
O1	0.9748 (2)	0.30053 (5)	1.0131 (2)	0.0538 (4)	
H1	0.9791	0.2855	0.9244	0.081*	
O2	1.10008 (19)	0.20185 (4)	0.70363 (18)	0.0444 (4)	
C1	0.8785 (2)	0.35871 (6)	0.7742 (3)	0.0344 (4)	
C2	0.9227 (3)	0.34426 (6)	0.9575 (3)	0.0393 (5)	
C3	0.9128 (3)	0.37438 (7)	1.0959 (3)	0.0474 (5)	
H3	0.9382	0.3637	1.2167	0.057*	
C4	0.8661 (3)	0.41905 (8)	1.0537 (3)	0.0513 (6)	
H4	0.8594	0.4386	1.1467	0.062*	
C5	0.8273 (3)	0.43664 (7)	0.8724 (3)	0.0451 (5)	
C6	0.7811 (4)	0.48343 (8)	0.8286 (4)	0.0652 (7)	
H6	0.7739	0.5032	0.9210	0.078*	
C7	0.7473 (4)	0.49996 (8)	0.6555 (5)	0.0782 (9)	
H7	0.7179	0.5309	0.6294	0.094*	
C8	0.7564 (4)	0.47067 (8)	0.5156 (4)	0.0739 (8)	
H8	0.7334	0.4823	0.3966	0.089*	
C9	0.7988 (3)	0.42509 (7)	0.5512 (3)	0.0542 (6)	
H9	0.8043	0.4061	0.4561	0.065*	
C10	0.8341 (3)	0.40654 (6)	0.7300 (3)	0.0388 (5)	
C11	0.8664 (3)	0.32609 (6)	0.6287 (3)	0.0355 (4)	
H11	0.8127	0.3352	0.5037	0.043*	
C12	0.9814 (3)	0.21388 (6)	0.5489 (3)	0.0329 (4)	
C13	0.9258 (2)	0.18457 (6)	0.3775 (3)	0.0317 (4)	
C14	0.8905 (3)	0.13760 (6)	0.3788 (3)	0.0348 (4)	
C15	0.8421 (3)	0.11196 (7)	0.2176 (3)	0.0471 (5)	
H15	0.8181	0.0807	0.2204	0.057*	
C16	0.8291 (3)	0.13237 (8)	0.0521 (3)	0.0529 (6)	
H16	0.7964	0.1148	−0.0565	0.064*	
C17	0.8642 (3)	0.17866 (7)	0.0464 (3)	0.0501 (5)	
H17	0.8559	0.1924	−0.0654	0.060*	
C18	0.9117 (3)	0.20441 (6)	0.2079 (3)	0.0396 (5)	
H18	0.9349	0.2357	0.2037	0.048*	
H2	0.799 (2)	0.2634 (6)	0.4086 (18)	0.047 (6)*	

### Atomic displacement parameters (Å^2^)

**Table:**

	*U*^11^	*U*^22^	*U*^33^	*U*^12^	*U*^13^	*U*^23^
Cl1	0.0587 (3)	0.0436 (3)	0.0540 (4)	−0.0002 (2)	0.0242 (3)	0.0116 (2)
N1	0.0399 (9)	0.0341 (8)	0.0326 (9)	0.0012 (7)	0.0095 (7)	−0.0065 (7)
N2	0.0402 (9)	0.0344 (8)	0.0278 (9)	0.0059 (7)	0.0039 (7)	−0.0035 (7)
O1	0.0788 (11)	0.0439 (8)	0.0389 (9)	0.0039 (8)	0.0224 (8)	0.0056 (7)
O2	0.0500 (8)	0.0404 (7)	0.0294 (8)	0.0053 (6)	−0.0006 (6)	0.0002 (6)
C1	0.0308 (9)	0.0349 (10)	0.0356 (11)	−0.0021 (8)	0.0105 (8)	−0.0039 (8)
C2	0.0366 (10)	0.0419 (11)	0.0401 (12)	−0.0046 (9)	0.0154 (9)	−0.0041 (9)
C3	0.0508 (12)	0.0563 (13)	0.0383 (12)	−0.0091 (10)	0.0205 (10)	−0.0107 (10)
C4	0.0491 (12)	0.0551 (13)	0.0530 (15)	−0.0102 (10)	0.0228 (11)	−0.0247 (11)
C5	0.0374 (11)	0.0402 (11)	0.0547 (14)	−0.0054 (9)	0.0141 (10)	−0.0149 (10)
C6	0.0664 (16)	0.0421 (13)	0.079 (2)	0.0021 (11)	0.0187 (14)	−0.0185 (13)
C7	0.092 (2)	0.0353 (13)	0.094 (2)	0.0088 (12)	0.0198 (18)	0.0016 (14)
C8	0.096 (2)	0.0482 (14)	0.0684 (19)	0.0107 (13)	0.0212 (15)	0.0119 (13)
C9	0.0691 (15)	0.0403 (12)	0.0506 (14)	0.0050 (10)	0.0196 (12)	0.0016 (10)
C10	0.0328 (10)	0.0354 (10)	0.0457 (12)	−0.0023 (8)	0.0120 (9)	−0.0045 (9)
C11	0.0353 (10)	0.0362 (10)	0.0318 (10)	−0.0013 (8)	0.0089 (8)	−0.0015 (8)
C12	0.0338 (10)	0.0324 (9)	0.0301 (10)	−0.0017 (8)	0.0093 (8)	0.0010 (8)
C13	0.0293 (9)	0.0341 (9)	0.0285 (10)	0.0028 (8)	0.0074 (8)	−0.0017 (8)
C14	0.0318 (9)	0.0339 (10)	0.0361 (11)	0.0023 (8)	0.0097 (8)	0.0005 (8)
C15	0.0470 (12)	0.0372 (11)	0.0528 (14)	−0.0003 (9)	0.0139 (10)	−0.0097 (10)
C16	0.0553 (13)	0.0575 (14)	0.0384 (13)	0.0062 (11)	0.0091 (10)	−0.0156 (11)
C17	0.0575 (13)	0.0597 (14)	0.0326 (12)	0.0077 (11)	0.0163 (10)	0.0002 (10)
C18	0.0434 (11)	0.0378 (10)	0.0355 (11)	0.0037 (9)	0.0124 (9)	0.0013 (9)

### Geometric parameters (Å, °)

**Table:**

Cl1—C14	1.737 (2)	C6—H6	0.9300
N1—C11	1.282 (2)	C7—C8	1.394 (4)
N1—N2	1.383 (2)	C7—H7	0.9300
N2—C12	1.347 (2)	C8—C9	1.368 (3)
N2—H2	0.896 (9)	C8—H8	0.9300
O1—C2	1.353 (2)	C9—C10	1.408 (3)
O1—H1	0.8200	C9—H9	0.9300
O2—C12	1.229 (2)	C11—H11	0.9300
C1—C2	1.388 (3)	C12—C13	1.493 (2)
C1—C10	1.443 (3)	C13—C14	1.394 (2)
C1—C11	1.445 (3)	C13—C18	1.395 (3)
C2—C3	1.402 (3)	C14—C15	1.375 (3)
C3—C4	1.354 (3)	C15—C16	1.375 (3)
C3—H3	0.9300	C15—H15	0.9300
C4—C5	1.411 (3)	C16—C17	1.377 (3)
C4—H4	0.9300	C16—H16	0.9300
C5—C6	1.415 (3)	C17—C18	1.379 (3)
C5—C10	1.418 (3)	C17—H17	0.9300
C6—C7	1.348 (4)	C18—H18	0.9300
			
C11—N1—N2	116.37 (16)	C8—C9—H9	119.6
C12—N2—N1	119.02 (15)	C10—C9—H9	119.6
C12—N2—H2	123.0 (13)	C9—C10—C5	118.02 (18)
N1—N2—H2	117.4 (13)	C9—C10—C1	122.90 (18)
C2—O1—H1	109.5	C5—C10—C1	119.08 (19)
C2—C1—C10	118.55 (17)	N1—C11—C1	121.25 (18)
C2—C1—C11	120.65 (17)	N1—C11—H11	119.4
C10—C1—C11	120.71 (17)	C1—C11—H11	119.4
O1—C2—C1	122.49 (17)	O2—C12—N2	122.65 (17)
O1—C2—C3	116.01 (18)	O2—C12—C13	123.31 (16)
C1—C2—C3	121.50 (19)	N2—C12—C13	114.02 (15)
C4—C3—C2	120.0 (2)	C14—C13—C18	117.70 (17)
C4—C3—H3	120.0	C14—C13—C12	123.07 (17)
C2—C3—H3	120.0	C18—C13—C12	119.22 (16)
C3—C4—C5	121.66 (19)	C15—C14—C13	120.87 (19)
C3—C4—H4	119.2	C15—C14—Cl1	117.58 (15)
C5—C4—H4	119.2	C13—C14—Cl1	121.53 (14)
C4—C5—C6	121.8 (2)	C16—C15—C14	120.25 (19)
C4—C5—C10	119.08 (19)	C16—C15—H15	119.9
C6—C5—C10	119.1 (2)	C14—C15—H15	119.9
C7—C6—C5	121.2 (2)	C15—C16—C17	120.3 (2)
C7—C6—H6	119.4	C15—C16—H16	119.8
C5—C6—H6	119.4	C17—C16—H16	119.8
C6—C7—C8	119.9 (2)	C16—C17—C18	119.4 (2)
C6—C7—H7	120.0	C16—C17—H17	120.3
C8—C7—H7	120.0	C18—C17—H17	120.3
C9—C8—C7	120.9 (3)	C17—C18—C13	121.45 (18)
C9—C8—H8	119.6	C17—C18—H18	119.3
C7—C8—H8	119.6	C13—C18—H18	119.3
C8—C9—C10	120.8 (2)		

### Hydrogen-bond geometry (Å, °)

**Table:**

*D*—H···*A*	*D*—H	H···*A*	*D*···*A*	*D*—H···*A*
N2—H2···O2^i^	0.90 (1)	1.97 (1)	2.842 (2)	164 (2)
O1—H1···N1	0.82	1.86	2.581 (2)	146

Symmetry codes: (i) *x*−1/2, −*y*+1/2, *z*−1/2.
